# Dementia and dignity of identity: A qualitative evidence synthesis

**DOI:** 10.1177/14713012211072929

**Published:** 2022-03-02

**Authors:** Cera E Cruise, Bonnie M Lashewicz

**Affiliations:** Department of Community Health Sciences, Cumming School of Medicine, 70401 University of Calgary, Calgary, AB, Canada

**Keywords:** dementia, dignity of identity, new materialism disability studies, caregivers, healthcare providers, long-term care

## Abstract

In the late stages of dementia, individuals rely on others for their wellbeing and this creates an ethical imperative for responsive dementia care. Through a qualitative evidence synthesis of literature on what constitutes responsive dementia care, we identified dignity of identity as a central theme. Dignity of identity is the status each of us holds in relation to others and reflects our past experiences and our aspirations for the future. We did a qualitative evidence synthesis of 10 qualitative studies conducted with a total of 149 research participants, 95 of whom had dementia, and 54 of whom were paid and family member caregivers to people with dementia. Using “new materialism disability studies” as our theoretical framework, we illustrate how environments, both material and discursive, shape the abilities of people with dementia in residential care settings (RSCs) to live well and we use our findings to point to ways forward in dignity of identity-enhancing dementia care practice. Echoing the literature, we observe that people with dementia have the virtual capacity to live with dignity of identity and illustrate how material conditions and discourse influence the transition of dignity of identity in people with dementia from a virtual capacity to an actual capacity and how demonstrated capacity in turn influences material conditions and discourse surrounding care for people with dementia in RSCs. We call for a greater acknowledgement within literature on dignity and dementia of structural barriers to dignity of identity-enhancing care. The COVID-19 pandemic has shown us the fatal consequences of insufficient material conditions in RCSs and we hope that on a societal level there is improvement to both the material conditions in RCSs as well as an improvement in discourse about those who live and work in RCSs.

## Introduction

As we age, we want the comfort of knowing that we will be treated with kindness and respect, that the person caring for us is gentle and sees us as a unique individual who has led a life of significance. We want to matter. Dementia affects an increasing number of people each year. In the late stages of dementia, individuals rely on others for their wellbeing and this creates an ethical imperative for dementia care that is responsive. Through our review of qualitative studies about what constitutes responsive dementia care according to those with dementia and their paid and unpaid caregivers, we identified the maintenance and promotion of what Nordenfeldt (2004) termed the “dignity of identity” as the foundational element. Dignity of identity is the status each of us holds in relation to others and reflects our past experiences and our aspirations for the future (Nordenfelt, 2004). It can be increased or violated by external actors and consequently, those with whom we interact have power over our conception of our self-worth. In this paper, dignity of identity is our foundation for challenging assumptions that people with dementia cannot exercise their own desires and our purpose is to conduct a qualitative evidence synthesis (Grant & Booth, 2009) to integrate and compare perspectives on responsive dementia care from the standpoint of people with dementia and their caregivers. We use this integration to illuminate the workings of dignity of identity as key to responsive care and to point to ways forward in dignity of identity-enhancing dementia care practice.

This qualitative evidence synthesis is fueled by reports from the literature about the importance of the dignity and personhood for people with dementia. We begin with a presentation of background literature on dementia and the relational workings of dignity of identity followed by a discussion of our theoretical framework for understanding the contextual nature of dignity of identity. Specifically, we adopt a theoretical framework of new materialism disability studies as outlined by Feely (2016) which we use to acknowledge that while all humans have the potential to be limited by the context in which they live, such limitation need not be permanent or irreversible. Our qualitative evidence synthesis methodology is then described followed by our review of qualitative studies of the care experiences of people with dementia in relation to their dignity. We conclude with a discussion of the ramifications and limitations of our findings, illustrating how a new materialism disability studies framing of dignity of identity can inform understandings of the necessary elements of responsive care for people with dementia. Kitwood and Bredin (1992) note the importance of a theory of dementia care, asserting that theory allows for “conscientization” that can become the basis of concerted action. Amidst elevated public interest in conditions of residential care for seniors given the tragic outcomes of the COVID-19 pandemic, we argue our qualitative evidence synthesis is as a timely reminder of the impacts that discourse and material conditions have on the lives of people with dementia.

### Background literature

People with dementia are at heightened susceptibility for having their identity underrecognized and indeed, violations of the dignity of identity of people with dementia occur as people with dementia are distinctly vulnerable to mistreatment by family members and paid caregivers given their increased reliance on these figures (Nordenfeldt, 2004; Palmer, 2013). Heggestad and Slettebø (2015) propose that vulnerabilities to being underrecognized and mistreated are tied to uncertainties that surround ideas of a sense of self in people with dementia which occur as a result of mistaken assumptions of personhood and identity as intrinsically related to intellectual cognition (Hughes, 2001). A large body of literature, including seminal works by Kitwood and Bredin (1992), Sabat and Harré (1992), and Kontos (2004) recognize that while an individual’s personality or life story cannot be changed, their environment and caregivers’ actions can be structured to promote—rather than disregard—their personhood (Mitty & Flores, 2007; Surr, 2006). Personhood and the dignity of identity are intertwined; when personhood is underrecognized, an individual’s dignity of identity is harmed (Higgins, 2014; Kitwood & Bredin, 1992).

Benner and Wrubel (1989) point out obstacles to promoting dignity of identity for people with dementia given that many people without dementia imagine dementia as a social death precursor to physical death. Social death is exemplified by Gove et al. (2015) who quote a physician research participant whose patients had dementia: “The fear’s about “am I going to be like this?” Am I going to end up like this? They need help doing this, that and the other and eventually they don’t recognize somebody. It’s living death” (Gove et al., 2015, p. 395). We counter that social death is not a function of ability to do for oneself and to recognize others, but rather social death occurs when others “cease to notice that the person is still alive, still has a world, still has concerns” (Benner & Wrubel, 1989, p.16 as cited in Palmer, 2013). In other words, social death occurs when dignity of identity is not supported by one’s environment (Mitty & Flores, 2007; Sabat & Harré, 1992). While the brain of a person with dementia is clinically harmed by the disease, the person does not have to be (Hughes, 2001).

The dignity of identity of people with dementia is promoted by an array of factors that comprise and support person-centered care including: familiarity with their environment; a sense of agency; a perception of belonging; valued roles and an occupation; a recognition of their life history; continuity; and feelings of comfort and security. As dementia advances, individuals’ need for these factors increase and change (Brooker, 2003; Cadieux et al., 2013; Kitwood & Bredin, 1992; Sabat and Harré, 1992). Through our qualitative evidence synthesis, we endeavor to consolidate perspectives on responsive dementia care from the standpoint of people with dementia and their caregivers, we examine studies about responsiveness of dementia care that provide in-depth, qualitative accounts from people with dementia and their caregivers, and we bring together their descriptions of person-centered practices that support dignity of identity and constitute responsive care.

### Theoretical framework

This paper is anchored in a new materialist view of disability—and we use disability to include impairments that accompany dementia—as a natural aspect of human existence that is both materially and discursively created (Feely, 2016). Discourse, the practice of using communicative actions (i.e. words, artwork, or gestures) to give meaning to social occurrences, can help us identify instances of low expectations of the capacity for personhood of people with dementia, while our theoretical anchoring in new materialism disability studies guides us to observe the consequences of negative discourse through the material condition of the corresponding deficiency of dignity of identity experienced by people with dementia (Fairclough et al., 2011). A change in the discourse of what it means to have dementia can change the material reality of having dementia and vice versa. By adopting a new materialist disability studies anchoring, we endeavor to organize and account for some of the forces that intersect in the experience of having dementia and we aim to influence discourses about the meanings of dementia.

**Figure 1.**
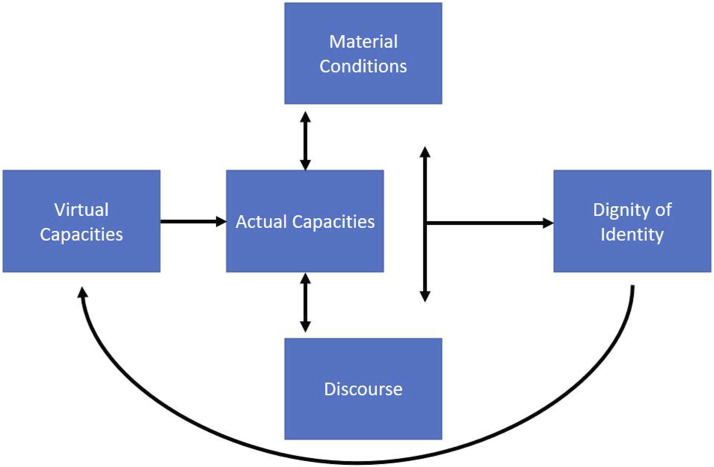
Theoretical framework of a new materialism disability studies understanding of dignity of identity.

Feely (2016), inspired by Deleuze, advances a new materialist disability studies anchoring by dividing capacities—for our purposes, human capacities—into actual capacities and virtual capacities. Actual capacities are actions that an individual is presently capable of doing, while virtual capacities are actions that an individual has the potential to do. Accordingly, a person with moderate to late stage dementia does not have the actual capacity to live independently but, provided with support that contributes to dignity of identity and reinforces sense of self, can live a fulfilling life. This is their virtual capacity and is well documented by literature on personhood and dementia (i.e. Kitwood & Bredin, 1992; Kontos, 2004; Sabat and Harré, 1992). When care reinforces the dignity of identity of people with dementia, people with dementia have an actual capacity to live fulfilling lives. We attempt to illuminate ways in which capacities are context dependent; a change in context (material reality) can change the capacity of people with dementia (thus shaping the discourse of dementia) and a positive discourse surrounding dementia can improve the material conditions of the facilities and communities where people with dementia live. Dignity of identity is a consequence of having both a positive discourse of dementia and the material conditions required to provide relational care to people with dementia. Discourse and material conditions are changed as actors form meaning out of the displayed capacity of an individual or population (in this case, people with dementia with dignity of identity). Thus, virtual capacities of people with dementia transition to actual capacities. In Figure 1 below, we visually depict the interplay of the phenomena represented by these theoretical concepts (Figure 1).

The foundations of dignity of identity consist of autonomy and integrity (Higgins, 2014; Nordenfeldt, 2004) and we overlay the new materialist concept of disability to guide thinking about the importance of material conditions and discourse in experiences of autonomy and integrity. Consistent with our understanding of people with dementia as capable of having dignity of identity with the support of their paid and unpaid caregivers, we view humans as social beings who cannot be defined in isolation of one another and we see autonomy as a relational, rather than individualistic, concept (Christman, 2004). A new materialist disability studies anchoring will support our effort to advance discourses of dementia and dementia care that are informed by people with dementia and their caregivers who supply evidence of what is entailed in treating people with dementia in autonomy supporting ways that honor their actions and promote their self respect or conception of integrity to yield dignity of identity (Stevens et al., 2013).

## Methods: Search protocol and data collection

In this qualitative evidence synthesis responsiveness in dementia care was our topic area and encompassed under responsiveness of care we included studies of dignity, continuity, and care in residential care settings (RCSs) and studies of professional practice in dementia care. We searched four databases on 20 October 2019: Academic Search Complete, CINAHL Plus with Full Text, Family and Society Studies Worldwide, and SociINDEX with Full Text using the search terms *autonomy or dignity* AND *dementia or Alzheimer’s* AND *long-term care or assisted living or nursing home or elderly home* AND *sense of self or identity or personhood*. This search yielded 90 articles; once duplicates were removed, we were left with 41 articles. These were narrowed to a sample of nine using the following inclusion criteria:

• ‘Types of articles’: only peer reviewed original research studies were included, and these had to present analyses of qualitative accounts of dementia care from which we could examine the workings of dignity of identity. Given our focus on dementia care broadly, end of life studies were excluded.
• ‘Types of participants’: studies had to include perspectives of people with dementia or their paid or unpaid caregivers who discussed experiences of dementia care and impact on the dignity of people with dementia.

One additional study was located through handsearching the reference lists of included studies. Data were collected by analyzing themes that lay across the findings presented in our included studies, which allowed us to observe a relatively broad understanding of the impact of our phenomena of interest (Grant & Booth, 2009), namely discourse and material conditions on the wellbeing of people with dementia as defined by the presence of dignity of identity. The table below (Table 1) provides a summary of the time, location, research design, and primary participants for each of our ten reviewed studies.

**Table 1.** Table of reviewed studies.

Title	Authors	Year	Where	Research design	Primary participants
Bedlam or bliss? Recognizing the emotional self-experience of people with moderate to advanced dementia in residential and nursing care	Godwin, B., & Poland, F	2015	United Kingdom	Interpretative Phenomenological analysis of in-depth interviews with ten people with advanced dementia (participants), and supplementary interviews with one family and one paid caregiver (interviewees) per participant with dementia to triangulate data	People with advanced dementia; ***n*= 13; 3 pilot participants and 10 participants**
Dignity and care for people with dementia living in nursing homes	Heggestad, A., Nortvedt, P., & Slettebø, A	2015	Norway	Phenomenological and hermeneutic perspective. Field observations of paid caregiver treatment of 15 people with dementia were recorded by the first author. Seven relatives were interviewed to gather relative perspectives on dignity and care	Relatives of people with dementia and researcher observations of staff interactions with people with dementia in nursing homes; ***n*=15 persons with dementia; *n*=7 relatives**
How individuals with dementia in nursing homes maintain their dignity through life storytelling—a case study	Heggestad, A. & Slettebø, A	2015	Norway	Phenomenological and hermeneutic perspective, case presented using narrative analysis of three stories gathered in a previous study (Heggestad, Nortvedt, & Slettebø, 2015). Data were gathered through participant observation and formal interviews	People with dementia; ***n*=3**
“It’s a Consolation”: The role of Christian religion for people with dementia who are living in care homes	Higgins, P	2014	United Kingdom	Interpretive Phenomenological analysis of semi-structured interviews with ten people with moderate to advanced dementia (7 female, 3 male)	People with moderate to advanced dementia (MMSE less than 20); ***n*=10**
Maturation of the MOUTh intervention: From reducing threat to relationship-centered care	Jablonski-Jaudon, R., Kolanowski, A., Winstead, V., Jones-Townsend, C., & Azuero, A	2016	USA	Observations of mouth care providers on the responses of 46 people with dementia to receiving oral care based on a theoretical framework of the neurobiology of threat perception	Mouth care providers observing people with dementia’s response to intervention; ***n*=46**
Continuity and change in life engagement among people with dementia	Kuosa, K., Elstad, I., & NormannH.K.	2014	Norway	Thematic narrative analysis of interviews with 11 relatives of people with dementia who either cared for their relative at home (*n*=3) or lived in RCS (*n*=7)	Relatives of people with dementia; ***n*=11**
Preserving personhood of individuals with advanced dementia: Lessons from family caregivers	Palmer, J	2013	USA	Hermeneutic phenomenological analysis of 3 in-depth interviews each with 15 participants who had placed a family member in a long-term care facility in the past 12 months	Family caregivers of a person with dementia; ***n*=15**
Dignity in people with frontotemporal dementia and similar disorders—a qualitative study of the perspective of family caregivers	Sagbakken, M., Naden, D., Ulstein, I., Kvaal, K., Langhammer, B., & Rognstad, M	2017	Norway	Qualitative, explorative, and descriptive study design conducting 11 semi-structured interviews with relatives of people with dementia	Relatives of people with dementia; ***n*=11**
Relational interaction. preserving dignity experience: Perceptions of persons living with dementia	Tranvåg, Petersen, & Nåden	2014	Norway	Application of Gadamer’s philosophical hermeneutics to transcriptions of interviews with eleven participants with mild to moderate dementia	People with dementia; ***n*=11**
Promoting a good life among people with Alzheimer’s disease	Zingmark, K., Sandman, P., & Norberg, A	2002	Sweden	Phenomenological hermeneutic interpretation of interviews with ten nurses working in special care units	Nurses; ***n*=10**

## Studies reviewed

### Findings

#### Participants in studies reviewed

Our ten studies encompass data collected from a total of 149 research participants. Of these, 37 had dementia and had been interviewed face to face. An additional 58 participants with dementia were considered research participants by study authors but had not been interviewed. These 58 are from Jablonski-Jaudon et al.’s (2016) study of 46 participants with dementia who received a mouth care intervention and whose responses were observed by mouth care provider participants (MCPs). The additional 12 participants were part of Heggestad et al.’s (2015) observation of 15 people with dementia going about everyday life in their care residence; 12 of these 15 are counted here and the remaining three were accounted for in the interview study of 37 participants with dementia (Heggestad & Slettebø, 2015) noted above. Thus, a total of 95 people with dementia informed the studies we reviewed. The remaining 54 participants were paid caregivers and family members who provided perspectives about dementia care responsiveness to dignity-needs through interviews and researcher observations. All but two reviewed studies gathered data exclusively from people with dementia, family members, or nurses in an RCS. The Tranvåg, et al. (2015) study of participants with mild to moderate dementia who were still living in their own homes is included as these participants provided detailed accounts of how interactions with others affected their dignity. The Kuosa et al. (2014) study is included because it offers perspectives from family members who were primary caregivers for someone with dementia still living in their own home (***n*=3)** or who played a significant caregiving role for someone with dementia living in an RCS (***n*=8)**.

Included studies were conducted in the United Kingdom, Norway, Sweden, and the United States of America. We begin with an overview of dimensions of dignity according to Tranvåg et al.’s (2015) research participants with dementia. We used these dimensions to sensitize our analysis of the studies reviewed which captured the simple and overarching idea that people value being treated like people. This encompasses being treated with kindness, respect, and empathy. We follow with the two major themes we generated to capture aspects of dementia care that influence dignity: 1–material conditions: food, clothing, and belongs, and 2–dignity promoting discourse. Our material conditions theme encompasses evidence of how food, clothing, and personal belongings can contribute to dignity of identity. Our theme of dignity promoting discourse is about non-tangible elements that contribute to dignity of identity and we open this theme with a description of how research participants characterize emotional space. We follow with evidence of three sub-themes comprising strategies for using emotional space to contribute to dignity of identity: a) engaging with residents’ stories and beliefs b) engaging with residents’ earlier identities and skills, c) connecting with and responding to residents’ rhythms: beyond a focus on tasks

### Preamble: Dimensions of dignity

Dignity is derived from acts of daily living; experiencing love, feeling appreciated, being included, treated with kindness, and having equal status with other human beings (Tranvåg et al., 2015). A participant with dementia spoke about his grandchildren and great-grandchildren looking up to him: “recognizing their love for you, is also a form of dignity… you feel they look up to you somehow. . . despite the diagnosis”(Tranvåg et al., 2015, p.582). Another participant with dementia portrayed how being needed can enhance dignity:

That they still need me. . . yes, the youngest (her grandchild) was preparing for his high school exam in German. . . and when he came in the door he called out: “Grandma!!“. . . You see, I have a degree in German. So we sat down. . . and worked hard. . . preparing for this oral exam. . . It’s good to be needed. (Tranvåg et al., 2015, p. 582, p. 582)

Family caregivers viewed acts as simple as paid caregivers saying ““Hello”, “How are you doing”, “Hang in there!”, pat you on the back, [and say] “You’re going to be okay” when walking by to be reassuring to people with dementia in RSCs as the deeper meaning is that the individual matters and is part of a community (Palmer, 2013, p.227). These findings illustrate the impact that the recognition of the humanity of a person with dementia can have on their ability to have dignity of identity.

## Material conditions: Food, clothing, and belongings

Physical spaces, defined as a compilation of tangible elements that we can see, taste, feel, smell, and hear, affect dignity in terms of how welcome or accepted we feel. They are a reflection of both material conditions and discourse. Family caregivers in Palmer’s (2013) study on preserving the personhood of people with advanced dementia expressed fear that their relative with dementia would cease to matter or cease to be seen as a person. For example, some reported visiting a RCS and finding that their relative had not been fed dinner or were cold and needing a sweater.

I found him in bed with a cold tray of food on his bedside table and this was like at 6:00 [in the evening] and the door [was] shut. Obviously nobody was going to feed him. So I went down to the nurse and said “Is anybody going to feed him?” And some aide came up and said, “I’ve already fed him.” (Palmer, 2013, p.226, p.226)

Another family caregiver spoke of a paid caregiver wanting to stop dressing her mother and instead use hospital gowns. This family caregiver pointed out the importance of clothing to her mother’s dignity, noting that if her mother were to be “out in the hallway in a gown or even a duster or a robe, she would be mortified” (Palmer, 2013, p. 226). Another invoked the loss of dignity that occurs when clothing is manipulated in the service of saving time:

They had a habit for a while of taking off her diaper and pulling down her pants and leaving them around her ankles [at nap time] and it bothered me. One time when I happened to be there when she was waking up from her nap, she was just squirming and moving all over that bed and frowning. I kept saying “Mom, what’s wrong?” “Nothing”. “Does something hurt?” “No” … I finally figured out that she’s trying to move her legs and can’t. Who of us would lay down for a nap and pull our pants down around our ankles and leave them there? None of us. (Palmer, 2013, p.226)

Along with food and clothing, personal belongings are important to dignity as reported by Zingmark et al. (2002) in their study of paid caregiver views on promoting a good life for people with Alzheimer’s disease. Paid caregivers noted the importance of being surrounded by personal belongings in order that a RCS may feel like home. Family caregiver participants in Kuosa et al.’s study of continuity and change in life engagement among people with dementia (2014) underline the importance of personal belongings:

We have tried to take the pictures she had at home, of the children and grandchildren. Yes, we have tried to arrange them in the same way they were at home. . . You cannot hang up pictures everywhere. It is not allowed. They have a kind of. . . wooden board or so. (Kuosa et al., 2014, p.215, p.215)

This daughter noted that the wooden board display space did not permit a reflection of her mother’s personality and lifelong love of photography, including having used the same camera she bought in the 1960s. The importance of personalized space is notable in Zingmark et al.’s (2002) RCS research site where each resident had their own bedroom decorated to their tastes and paid caregivers indicated that photographs provided a sense of the residents’ histories. Sagbakken et al. (2017) further demonstrate the importance of personalizing space as key to dignity noting a family caregiver described a lack of personalization at his wife’s RCS: “She has had relatives visiting her […] and they are shocked to see her there”; correspondingly, Sagbakken et al.’s (2017) family caregiver participants described RCSs as a ‘closed system’ that did not allow for individualization. Some family caregivers pointed to locks on doors and lack of personal items as representing a social death (Sagbakken et al. 2017). One daughter compared moving her mother into the RCS to “delivering her into a … into a deposit box, and technically it was similar to her being dead” (Sagbakken et al., 2017, p.7).

At the same time, Godwin and Poland (2015), who studied the emotional self-experience of people with dementia, raise potential for personalizing of RCS space to trigger negative feelings. One participant with dementia in particular had thrown mementos of his Church and his career out his bedroom window. Godwin and Poland hypothesized that these mementos represented a contrast between past accomplishments and an unhappy present that were too painful to tolerate (2015).

## Dignity promoting discourse

The studies reviewed typically included reference to the importance of elements of space that are not tangible or quantifiable and that contribute to dignity of identity for people with dementia. Researchers note that to “be” a person requires being treated as a person. Participants with dementia articulated wanting to be treated in a manner that respected their choices (Godwin & Poland, 2015; Tranvåg et al., 2015) while family caregivers spoke in terms of paid caregivers going beyond physical care in order that people with dementia can be “taken seriously as a human being” (Heggestad et al. 2015, p.833). Jablonski-Jaudon et al. (2016) built their intervention study on the goal that “every individual in the caring relationship should experience a sense of worth, purpose, and achievement” (p.17).

A family member participant in the study by Heggestad et al. (2015) illustrated how important it was to her that her mother’s caregivers had a dignity of identity promoting ethos: “She’s respected as the person she is; they don’t treat her as a ‘demented person’ in a wheelchair who isn’t able to move” (p.833). Correspondingly, a participant with dementia in Tranvåg et al.’s study described her self-satisfaction as contingent on others: “When those you meet don’t acknowledge you as an equal. . . making you feel small and unimportant [. . .] when they are superficial. . . I can feel the difference” (2015, p.584). An emotional space where people with dementia feel respected and important is created when paid caregivers invite and engage with stories and beliefs of residents with dementia and drawing upon insights offered by family members of residents with dementia.

### a. engaging with residents’ stories and beliefs

In their study of emotional self-experience of people with moderate to advanced dementia in RCSs, Godwin and Poland (2015) asked participants how they make sense of their personal and social worlds. Participants shared imagined stories in which they described assuming roles including that of a decorated French army veteran, a successful property investor, a heroic nurse, and for two participants, being members of the Japanese Secret Service. While Godwin and Poland did not verify whether these stories were connected to residents’ past lives, these authors point out that the roles described through the assumed imaginary stories were all status enhancing and may have provided satisfaction amidst an environment with minimal stimulation (Godwin & Poland, 2015).

Heggestad and Slettebø (2015) studied how dignity is maintained through storytelling and they conducted a case study of three people with dementia in RCS. One participant responded in similar fashion to participants in Godwin and Poland’s (2015) study as she described having been an adored singer and attracting crowds to the restaurant where she worked, thus casting herself in an active role rather than as “a passive resident who sat sleeping in her chair waiting for the next meal” (Heggestad & Slettebø, 2015, p. 2327). Heggestad and Slettebo (2015) reported that their other two participants shared stories during which they appeared to calm, reclaim identity, and project themselves as experts (p. 2324).

Affirmation of dignity was a goal of Jablonski-Jordan et al.’s (2016) mouth care intervention study with residents resistant to mouth care and engaging with resident’s stories as a means of ‘entering the individual’s reality’ was part of the intervention. For example, when residents expressed having already brushed their teeth, mouth care providers would agree but would suggest that because the resident had eaten dinner, it would be best to brush again. Similarly, Zingmark et al. (2002) reported on how engaging with resident’s stories created a supportive emotional space that enhanced wellbeing of people with Alzheimer’s disease. Paid caregivers reported orientating their actions towards the comfort of the resident and working within the resident’s reality: “Even if their talking sounds confusing to me, it’s of importance to them. Often I think they are dealing with old events that are not finished in their minds” (Zingmark et al., 2002, p.53).

Engaging with residents’ religious beliefs is a further component of emotional space through which caregivers can promote residents’ identity as members of a community (Higgins, 2014). Higgins interviewed ten people with moderate to advanced dementia about the role of Christian religion in their lives. Participants described their faith as a “part of me”. A higher power, in this case “God,” gave participants a sense of meaning and orientation. One participant said: “Well, I, I feel that He’s concerned about. . . everybody and. . . me, as an individual as well” (2014, p.329). Higgins concluded that for people with dementia, faith can provide “an anchor in the sea of confusion” (2014, p.332).

### b. engaging with residents’ earlier identities and skills

Zingmark et al. (2002) found that becoming familiar with residents’ life histories allowed paid caregivers to understand the residents’ identities and skills and in turn, provide care that promoted belonging and dignity. Paid caregivers reported having visited previous homes and places of significance to residents to enhance their understanding of residents as whole people. Paid caregivers were then more able to “read between the lines” to understand residents’ behavior (Zingmark et al., 2002). Similarly, in Jablonski-Jaudon et al.’s (2016) mouth care intervention study, through regular contact, mouth care providers gained knowledge of the life stories of residents that allowed them to place themselves in the residents’ reality. For example, one resident who refused to let anyone brush his teeth was a former teacher. The former teacher’s mouth care provider told him that his students were waiting, thus prompting the former teacher to allow the mouth care provider to brush his teeth and be ready to meet his responsibilities as a teacher.

Using data from their observational study at two RCSs, Heggestad and colleagues (2015) highlight how paid caregivers can engage with residents’ earlier identities and skill (Heggestad et al., 2015; Heggestad & Slettebø, 2015). These researchers report how a paid caregiver facilitated a resident’s participation in a lunch time conversation by drawing on knowledge of the resident. In this case, the paid caregiver brought up “puttees,” a type of legging which the resident wore as a child in going to school in wintertime. No one at the RCS had known what puttees were and puttees became a topic in which the resident was the expert (Heggestad et al., 2015).

Zingmark et al. (2002) reported that paid caregivers used knowledge of skills residents had used in earlier life to support residents in making contributions to their RCS and made claims to the effect of: “I think one feels well when still able to do things. That gives self confidence” (Zingmark et al., 2002, p.53). This practice of supporting resident contributions is illustrated in the study by Sagbakken et al. (2017) where a family member described paid caregivers as follows: “They try to engage him, as when they told me that they had received some new furniture and he sat there and worked and screwed it together, and after a while he took over and completed the whole thing” (Sagbakken et al., 2017, p.7). Conversely, family caregiver participants in Kuosa et al.’s (2014) study on continuity and change reported that a lack of opportunities for their relative to do tasks such as preparing meals led to a quick transition from being active to passively waiting for caregivers to provide for them. Godwin and Poland (2015) underline people with dementia as desiring to be contributors and “wishing to act as agents for good”; these researchers describe how a participant with dementia who had a “cognitive score equivalent to zero”^1^ showed concern for her paid caregivers while being observed by researchers by advising caregivers to “not overdo it and hurt themselves” (Godwin & Poland, 2015, p.242).

### c. connecting with and responding to residents’ rhythms: beyond a focus on tasks

Paid caregivers spoke of appreciating and responding to residents’ sense of reality moment to moment. A paid caregiver in the study by Zingmark et al. (2002) claimed: “It’s the present moment that counts, that’s how I reason. The main thing is that the situation is good when experiencing it, even if soon forgotten…I have to adjust to their rhythm” (p.53). Another paid caregiver in the study by Zingmark and colleagues pointed to the importance of connecting with residents beyond care task interactions: “It’s not always about doing things. An important part of the care is the community. We sit together, and they can rest. They feel well” (p.53).

A family caregiver participant in Heggestad et al.’s (2015) study also captured the importance of connecting and responding to residents in ways that transcend a focus on care tasks as she described desirable characteristics of a paid caregiver: “Someone who is calm. Gentle, and has time to sit down. . . listening. I actually think that’s the most important thing they can give. If they manage to help them with showering, I don’t think that’s the most important thing” (p. 833). Correspondingly, in her description of what care ought to look like for her father with dementia, one of Palmer’s (2013) family caregiver participants explained “To me, it isn’t just about giving them meds. It is about how they are making them feel that they’re still valuable and they’re still loved, and somebody really cares” (p.227). Participants in Tranvåg et al.’s (2015) study echo the importance of connecting with and responding to residents with dementia who were “allowed to be the center of attention” during interactions (p.584). Paid caregivers saw this as a demonstration of respect for residents that counteracted the stigma of dementia.

Some participants in our reviewed studies illustrated the theme of connecting with and responding to residents beyond a focus on care tasks by describing the use of touch. Touch can be a tangible means for creating emotional space and examples included hand holding and rubbing someone’s upper back if they appear comfortable (Jablonski-Jaudon et al., 2016; Palmer, 2013). The wife of a resident with dementia pointed out that “You can just hold his hand, and then sometimes he’ll light up like a Christmas tree” (Palmer, 2013, p.227).

Contrastingly, Jablonski-Jaudon et al. (2016) caution that touch can be perceived as restraining or forceful. A family caregiver in Palmer’s study (2013) was troubled by how touch was used forcefully to administer medications: “like in I guess pudding or something and it’s … well I wouldn’t want it shoved into my mouth like that” (Palmer, 2013, p. 227). A family caregiver in the study by Heggestad et al. (2015) described a high level of responsiveness as difficult given that paid caregivers often did not have time to connect with residents outside of care tasks: “They are running from one thing to another... they can’t sit down, one to one. (. . .) So it’s a question of resources” (p.834).

## Discussion

Because dignity of identity is derived from external actors, and people with dementia are reliant on others for care as their disease progresses, our analysis of the dementia care literature in terms of how care affects the dignity of identity of people with dementia is a striking illumination of the importance of the relational workings of care. By using a new materialist framing of dignity of identity, we combine our focus on relational care with a focus on the material elements of care and how such elements inform and are informed by discourse of dementia. We highlight the relational adeptness of caregivers who draw on the life stories and beliefs of people with dementia in ways that support dignity of identity.

### Discourse

Caregivers in the reviewed studies went along with stories and beliefs expressed by residents and as such, supported resident wellbeing over an expectation that residents to be oriented to factual realities. Through such communicative actions in support of the dignity of identity of people with dementia, caregivers reflected a positive discourse of dementia. When material conditions supported residents to continue to process events in accordance with their own realities and thus, make meaning of their lived experience, residents’ virtual capacity for dignity of identity was transformed to an actual capacity. This “going along with” strategy runs counter to accuracy driven, western ideals and warrants greater recognition as a distinct, dignity of identity-enhancing skill employed within dementia care settings.

Caregivers who supported dignity of identity also tapped into residents’ earlier identities and skill sets including by centering conversation on things used by residents in their earlier lives or by enlisting assistance from residents in performing tasks that required skills residents had developed earlier in their lives. Such tapping into resident identities and skill sets, along with embracing residents’ stories and beliefs, allowed for caregivers to reveal dignity of identity relevant insights into the often-hidden world in which people with dementia exist. Sharing our life story humanizes us to others. We are no longer representative of a group or illness; we are our own individual with a unique narrative who is worthy of respect.

### Material conditions

Physical realities provided essential contexts for, and interface with, relationally focused care. Material elements in our review included food, clothing, and belongings. The profound impact of such elements was underlined by the daughter who found her mother forced to nap with her pants around her ankles (Palmer, 2013); this example leaves a haunting worry that caregivers may have only superficial insight into the personality of people receiving care and/or may be so rushed in their work that a person receiving care would be left in such an undignified position. Indeed, being left to nap in such a position makes this mother appear socially dead in alignment with Benner & Wrubel’s (1989) definition of social death. Missing from the literature were discussions on more structural material conditions such as funding practices, staffing numbers, and family access to RCSs. Structural deficiencies in policies governing residential care settings prior to and during the COVID-19 pandemic—such as insufficient staffing ratios—impact the quality of life of all residents in RCSs and may explain the resulting maltreatment of residents such as the one described above in Palmer’s (2013) study (Hirdes et al., 2020).

Touch comprised a further, double-edged, physical element that could be used either to reassure and “light up” a person’s day, or to coerce a person in interactions such as those related to administering medication. As such, we highlight the intertwining of material and discourse-informed elements of care and through this bringing together of material conditions and discourse, we respond to Kontos and Martin (2013) who critique dementia literature as being polarized and focused either on the biological effects of dementia, or as “privileging of the social over the corporeal” as part of the ‘personhood’ movement (Kontos & Martin, 2013, p.289).

### Actual capacities

We illustrate that dignity of identity is an outcome of the intertwining of material and discourse-informed elements of care of people with dementia. The result is that the virtual capacity of people with dementia to live well becomes an actual capacity, reinforcing positive dementia discourses in the form of resistance to the assumption that people with dementia experience profound harm to their dignity of identity as a symptom of their dementia. Indeed, as well as presenting evidence from research participants with dementia who expressed feeling having changed with age, but not experiencing this change as inherently negative (Godwin & Poland, 2015), we present evidence of relational care taking place in material contexts that allow a response to residents beyond a focus on tasks and can leave people with dementia able to “rest” and feel well”.

Brooker (2003) frames supporting person-centered care for people with dementia as a Goldilocks’ problem. There cannot be “too little” or “too much” emphasis on elements of care, the balance must be “just right.” An overly positive discourse of dementia becomes a sort of “care evangelism” which cannot be met without material conditions such as training for caregivers or sufficient architectural design in RCSs. Correspondingly, a plethora of material resources are of limited use if such resources are directed towards framing dementia as a medicalized problem to be solved. An example of a potential victim of “care evangelism” from our findings was the man who belonged to the Church who threw his belongings out of the window; it was not clear that anyone had asked if *he* wanted those mementos in his room, and while they were displayed with presumed good intentions, the gesture amounted to an unwanted platitude (Brooker, 2003; Godwin & Poland, 2015).

This review of dignity of identity-enhancing care practices was conceived of well before the COVID-19 pandemic struck. Yet our findings have compelling meaning in the evolving context of the pandemic and we are struck by the relevance of how care practices that support dignity of identity for people with dementia are the very same practices recommended by public health officials dedicated to counteracting the tragic effects COVID-19 has held for RCSs. Our most pervasive finding is about the dignity of identity supporting power of continuity in relationships between residents with dementia and their caregiving staff and the attendant coherence of life history such relationship continuity affords. COVID-19 is a stark illumination of the consequences of staffing inconsistencies and shortages in RCSs which, while amplified by COVID-19, is a longstanding concern (Holroyd-Leduc & Laupacis, 2020; White et al., 2020). As COVID-19 continues to exact its toll on RCSs, we close with a single, overarching recommendation that every policy effort possible be made to promote staff continuity in RCSs in the dual service of optimizing both infection prevention and dignity of identity protection.

### Limitations

Through this review of dignity of identity-enhancing dementia care practices, we endeavored to practice critical allyship and stand in solidarity with people with dementia (Nixon, 2019). Key to this effort was our consolidation of perspectives from the standpoint of people with dementia and their caregivers. Yet, the perspectives of people with dementia that we reviewed are limited given that these people with dementia had the ability to verbally communicate. Thus, we do not include perspectives from people with dementia who cannot verbally communicate. Further, the studies included in this review were all conducted in high income western countries, notably Nordic countries. The absence of studies with participants from low and middle income countries who live in a different material reality and have different discourses surrounding dementia is a significant limitation of our work. Nonetheless, we argue that framing care for people with dementia as a material reality advances understandings of dignity of identity can contribute to a discourse of people with dementia as full people, which in turn reinforces the idea that treatment ought to preserve their dignity of identity.

## Notes

1 Godwin and Poland used both the 6CIT test and Guy’s Advanced Dementia Schedule to ascertain how advanced their participants’ dementia was, but neither the 6CIT test nor Guy’s Advanced Dementia Schedule measure cognitive score (Gale & Larner, 2017; Ward et al., 1993). However, both measures have been compared to the Mini-Mental State Exam (MMSE), which does give a cognitive score. Guy’s Advanced Dementia Schedule is used to further differentiates between individuals who have a zero on the MMSE, perhaps explaining how Godwin and Poland came to an understanding that their participant had a “cognitive score equivalent to zero.”
